# *VdPAT1* encoding a pantothenate transporter protein is required for fungal growth, mycelial penetration and pathogenicity of *Verticillium dahliae*

**DOI:** 10.3389/fmicb.2024.1508765

**Published:** 2025-01-17

**Authors:** Stephen Mwangi Kamau, Yongtai Li, Tiange Sun, Feng Liu, Qian-Hao Zhu, Xinyu Zhang, Jie Sun, Yanjun Li

**Affiliations:** ^1^The Key Laboratory of Oasis Eco-Agriculture, Agriculture College, Shihezi University, Shihezi, Xinjiang, China; ^2^CSIRO Agriculture and Food, Canberra, ACT, Australia

**Keywords:** cotton, *Verticillium dahliae*, RNA-Seq, pantothenate transporter, mycelial penetration

## Abstract

**Introduction:**

The soil-borne vascular fungus *Verticillium dahliae* is a phytopathogenic fungus known to attack cotton crop causing Verticillium wilt. In previous study, we identified a pantothenate transporter gene (*VdPAT1*) in *V. dahliae* which can be induced by root exudates from a susceptible cotton variety.

**Methods:**

In this study, we generated *VdPAT1* deletion mutants and complementary strain via homologous recombination by a PEG-mediated transformation method and used for the gene functional characterization.

**Results and discussion:**

The *VdPAT1* deletion mutants displayed reduced colony growth, melanin production, spore yield and germination rate, showed abnormal mycelial branching and decreased ability of mycelial penetration and utilization of nutrients (carbon, amino acids and vitamin), leading to a lower pathogenicity. Comparative transcriptome analysis of wild-type and mutant strain cultivated on sterilized carboxymethyl cellophane membranes found that the amino sugar and nucleotide sugar metabolism pathway, which was related to chitin synthesis and degradation as well as UDP-glucose synthesis, was the most significantly down-regulated pathway in *VdPAT1* deletion mutant. Chitin and β-1,3-glucan content determination found that the chitin content in *VdPAT1* deletion mutants was significantly lower, while β-1,3-glucan content was higher than that of wild-type and complementary strains. The ratio change of chitin and β-1,3-glucan content in *VdPAT1* deletion mutants might lead to abnormal branching of mycelium, resulting in the reduced penetration ability of *V. dahliae*. The decreased chitin content in *VdPAT1* mutants impaired the fungal cell wall integrity, leading to their increased sensitivity to external stresses.

**Conclusion:**

Together, the results demonstrated that *VdPAT1* is required for growth, development, resistance to external stresses, mycelial penetration and pathogenicity of *V. dahliae*.

## 1 Introduction

Cotton (*Gossypium hirsutum* L.) is an economically important crop and a major source for organic fiber worldwide (Liu et al., 2022). Like other valuable crops, cotton is often subjected to various biotic and abiotic stresses. Verticillium wilt (VW) disease, mainly caused by *Verticillium dahliae*, brings a major threat to successful commercial cotton production globally. It has been reported that this disease can lead to significant reduction in fiber yield and quality, resulting in huge economic losses every year (Song et al., 2020). *V. dahliae* is a soil-borne phytopathogenic fungus that can survive in the soil for many years as long-living dormant microsclerotia (Wilhelm, 1955), making it difficult to control. Exploring the key genes and molecular mechanisms involved in the pathogenic process of *V. dahliae* is crucial for developing effective strategies for the consistent management of VW.

The microsclerotia of *V. dahliae* germinates towards the roots following inducement by the root exudates from host plant and produces infectious hyphae (Klimes et al., 2015), which enters the plant through the root tip, passes through the root cortex, and finally colonizes the xylem vascular tissues, causing disease symptoms in plants, including wilting, leaf chlorosis, leaf shedding, vascular tissues necrosis, growth retardation and even death (Klosterman et al., 2009). During the infection process, pathogens need to utilize various exogenous metabolites secreted by the host to obtain nutrients and energy. Vitamins are important nutritional components for living organisms that can ensure normal tissue growth and development (Matherly and Hou, 2008). Vitamin transporter genes are used to obtain vitamin nutrients from the host plants which are absorbed via the hyphae for metabolism (Wilson et al., 2019). Only a few vitamin transporter genes have been identified in *V. dahliae*. Gene knockout or silencing of vitamin transporter genes (*VdThit4*, *VdThit*, and *VdTI20*) led to slower growth and development, as well as reduced pathogenicity of *V. dahliae* (Jurgenson et al., 2009; Qi et al., 2016; Qin et al., 2020). More vitamin transporter genes in *V. dahliae* need to be identified due to their significance role in vitamin nutrient uptake.

Pantothenate (vitamin B5) is the key precursor to the essential co-factor coenzyme (CoA), and participates in many essential biological and cellular processes in living organisms (Gihaz et al., 2022). It has been reported that pantothenate is essential for vegetative growth and development of fungi (Ipcho et al., 2012). Fungal organisms either take up pantothenate from the medium or synthesize it *de novo*, but they mainly acquire it externally (Olzhausen et al., 2009). In fungi, functional pantothenate transporter have become a useful means of vitamin B5 conveyance in the plasma membrane of the fungus (Stolz and Sauer, 1999). The high affinity vitamin B5 transporters belong to the major facilitator superfamily (MFS) and are involved in transportation of exogenous pantothenate into cells by use of hydrogen symporters (Stolz and Sauer, 1999). Several pantothenate transporter genes have been identified for fungi. For example, a pantothenate transporter gene liz1^+^ in *Schizosaccharomyces pombe* was found to be required for extracellular pantothenate uptake (Stolz et al., 2004). In *Cytospora chrysosperma*, a pantothenate transporter gene *CcPtc1* was found to be necessary for carbohydrate synthesis and virulence (Yang et al., 2023). However, pantothenate transporter gene has not yet been identified in *V. dahliae*.

In our previous study, we performed transcriptome analysis using RNA-sequencing (RNA-seq) to investigate gene expression of *V. dahliae* after induction by root exudates from cotton varieties with different resistance responses to the pathogen. It was observed that the expression level of a pantothenate transporter liz1 gene (VDAG_02269) was significantly up-regulated after sensing the root exudates from susceptible, tolerant and resistant cotton varieties (Zhang et al., 2020), implying that vitamin transportation is necessary for the infection process of *V. dahliae*. In this study, we obtained *VdPAT1* deletion mutants and analyzed the function of this gene in the growth, development and pathogenic process of *V. dahliae*. We then performed comparative transcriptome analysis using RNA-seq and compared the transcript profiles between mutant strain (∆*VdPAT1*) and the wild-type (Vd592). Based on the transcriptome analysis, a better understanding of transcriptional changes in the *VdPAT1* mutant strain during penetration process was obtained. This study helps understand the molecular mechanisms associated with fungal penetration and provides appropriate strategy for effective management of VW in cotton.

## 2 Results

### 2.1 Bioinformatics analysis of *VdPAT1* gene

**Figure 1:**
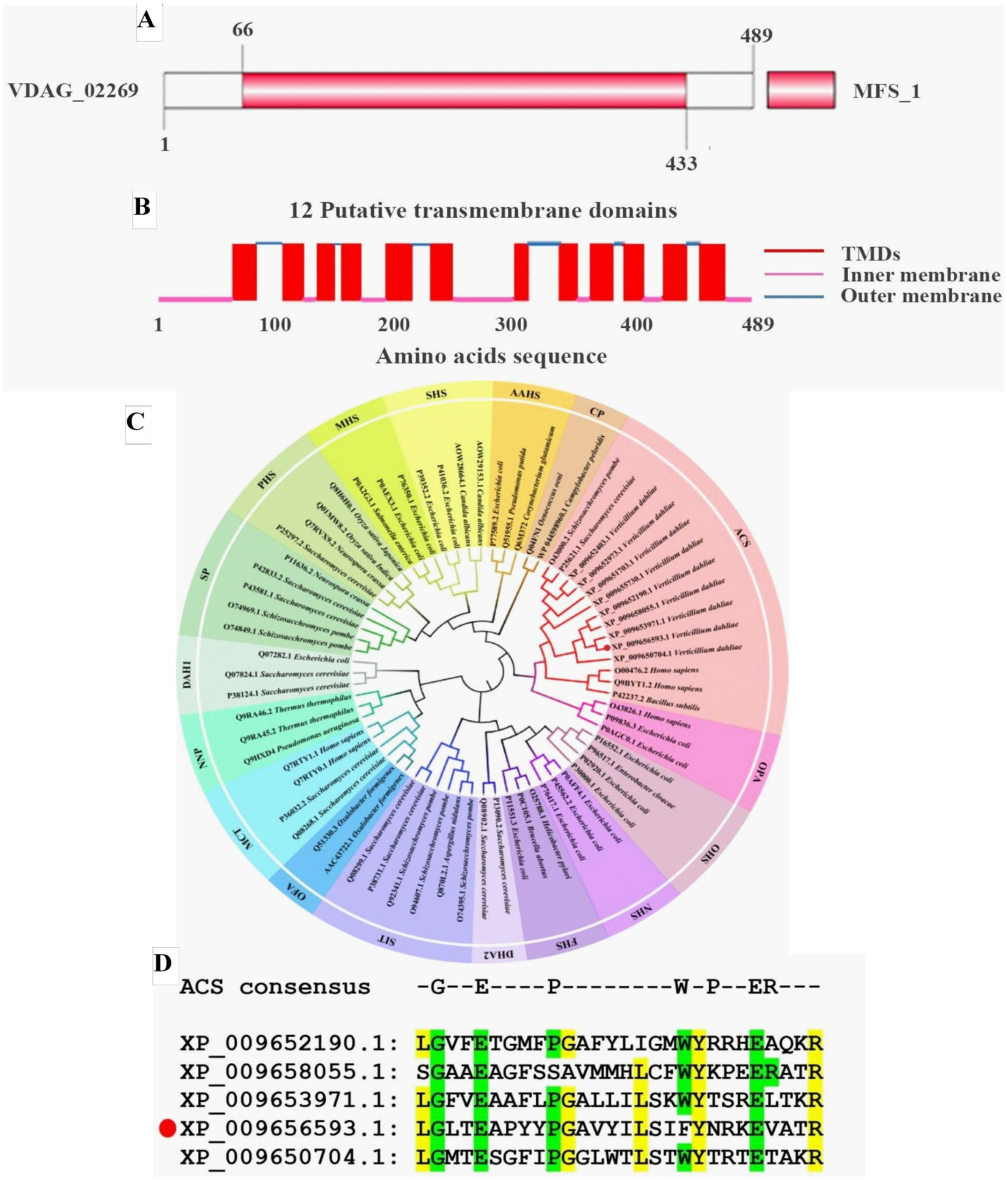
Bioinformatics analysis of pantothenate transporter genes. **(A)** The MFS conserved domain in VdPAT1 predicted by SMART tool. **(B)** The transmembrane domains (TMDs) in VdPAT1 predicted by Deep TMHMM software. **(C)** The phylogenetic tree for pantothenate transporter genes from *V. dahliae*, other fungi and bacteria. The tree was generated using MEGA 11.0 with the neighbor-joining method and 1,000 bootstraps replications. **(D)** Multiple sequence alignment showing the conserved residues in pantothenate transporters of ACS subfamily. “GEPWPER” are the reported conserved residues in ACS subfamily. The yellow colour illustrated the conserved residues of 5 members of ACS subfamily. The protein (XP_009656593.1) marked with a red circle is VdPAT1.

In our previous RNA-seq analysis, we found that the expression level of a pantothenate transporter liz1 gene (VDAG_02269) increased by more than 4-folds upon sensing root exudates from a susceptible cotton variety (Zhang et al., 2020). It was assumed that this gene plays important role in *V. dahliae*-cotton interaction and is required for the pathogenicity of *V. dahliae*. Therefore, it was selected as a candidate for functional study. The VDAG_02269 was designated as *VdPAT1*, which contained a predicted open reading frame of 1,470 bp, encoding 489aa residues. Structural prediction found that VdPAT1 harbored a major facilitator superfamily (MFS) domain from 66 to 433aa (Figure 1A), suggesting that *VdPAT1* belongs to MFS superfamily. A total of 12 putative transmembrane structural domains (TMDs) were identified in VdPAT1 (Figure 1B). Phylogenetic analysis of VdPAT1 protein with other 67 protein sequences from MFS superfamily revealed that VdPAT1 belongs to anion/cation symporter (ACS) subfamily (Figure 1C). Multiple sequence alignment of VdPAT1 with four other members from ACS subfamily revealed that they all contain 7 conserved residues in their fourth transmembrane domain (Figure 1D).

### 2.2 *VdPAT1* is required for the colony growth and melanin formation of *Verticillium dahliae*

**Figure 2:**
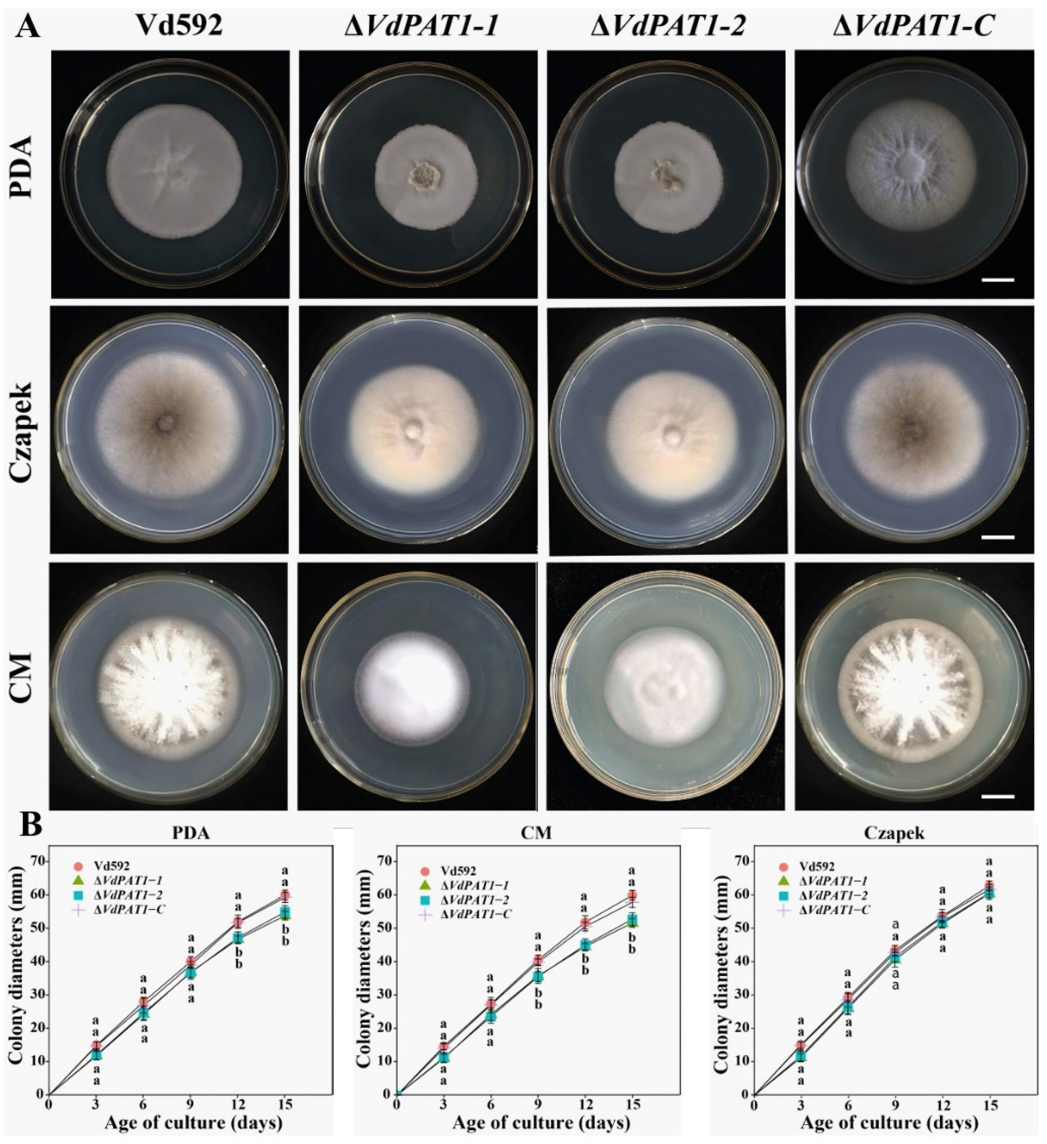
Colony morphology of different *V. dahliae* strains. **(A)** Colony morphology of different *V. dahliae* strains on PDA, Czapek and CM medium plates. Images were taken at 15 days post cultivation. **(B)** Colony diameters of different *V. dahliae* strains on PDA, Czapek and CM media plates. Scale bars represent 10 mm. Data were statistically analyzed on R environment (version 4.3.2). The ggplot2 package was employed to generate bar plots which represented mean ± standard deviation from three independent repeats. Significance differences between treatments were analyzed by one-way ANOVA using Duncan’s multiple range tests (DMRT) implemented on agricolae package. Different letters on error bars represent significance differences at *p* ≤ 0.05.

*VdPAT1* deletion mutants were generated by replacing partial sequence of *VdPAT1* with the hygromycin resistance gene (HPH) gene. A functional *VdPAT1* ORF was introduced into the *VdPAT1* mutant to generate complementary mutant strain. Finally, two *VdPAT1* deletion mutants (Δ*VdPAT1-1* and Δ*VdPAT1*-*2*) and one complementary mutant strain (Δ*VdPAT1*-*C*) were obtained and confirmed by PCR and qRT-PCR (Supplementary Figures S1A,B). To investigate the effects of *VdPAT1* on the fungal growth, the wild-type (Vd592), deletion mutants (Δ*VdPAT1*-*1* and Δ*VdPAT1*-2), and complementary mutant strain Δ*VdPAT1*-C were cultivated on three different media (PDA, CM (complete medium) and Czapek), and their colony diameters were measured every 3 days (Figure 2A). It was found that Δ*VdPAT1*-*1* and Δ*VdPAT1*-*2* mutants had a significant reduction in colony diameters as compared to Vd592 and Δ*VdPAT1*-*C* strains on PDA and CM media, while there were no significant differences in colony diameters among all strains on Czapek media (Figure 2B). We also found that Δ*VdPAT1*-*1* and Δ*VdPAT1*-*2* mutants did not produce melanin as compared to both Vd592 and Δ*VdPAT1*-*C* strains when cultured on CM and Czapek media (Figure 2A). These results suggested that *VdPAT1* is involved in colony growth and melanin formation, and its deletion results in lower growth rate and no melanin production of *V. dahliae*.

### 2.3 *VdPAT1* is required for the conidial production, germination, mycelial growth and penetration of *Verticillium dahliae*

**Figure 3:**
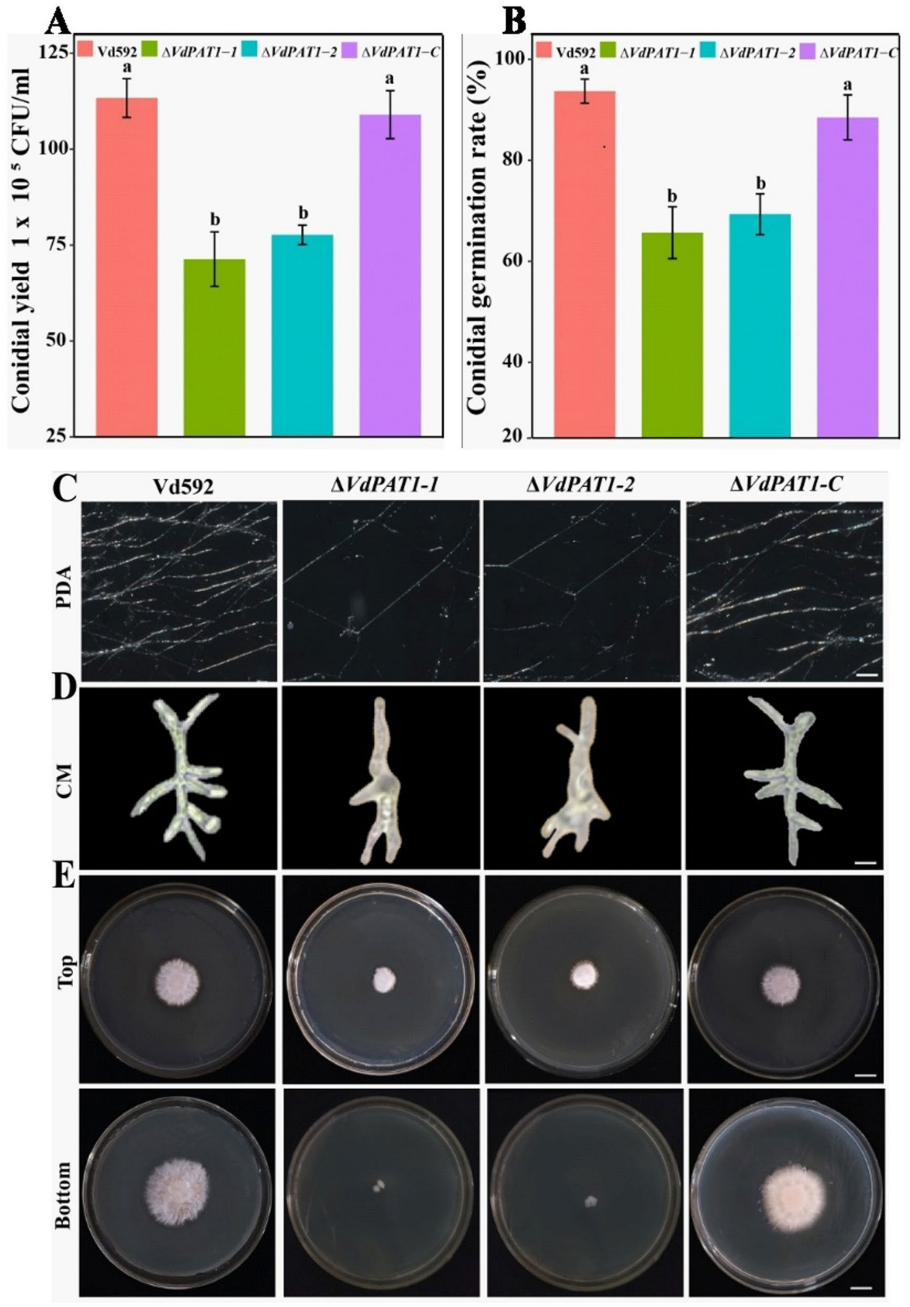
Conidial yield, conidial germination rate, and mycelial growth, morphology and penetration abilities of different *V. dahliae* strains. **(A)** Conidial yield of different *V. dahliae* strains after 7 days of culture at 25°C in liquid CM medium. **(B)** The conidial germination rate of different *V. dahliae* strains after 6 h of incubation at 25°C in liquid CM medium. **(C)** Mycelial growth of different *V. dahliae* strains after 5 days of culture on PDA medium plates. Scale bars represent 100 μm. **(D)** Mycelial morphology of different *V. dahliae* strains after 30 h of incubation at 25°C in liquid CM medium. Scale bars represent 100 μm. **(E)** Cellophane penetration assay of different *V. dahliae* strains. Different strains were grown on PDA medium plates covered with cellophane at 7 days post cultivation (Top). Different strains were gown on PDA medium after removing cellophane for another 7 days of cultivation (Bottom). Scale bars represent 1 cm. Data were statistically analyzed on R environment (version 4.3.2). The ggplot2 package was employed to generate bar plots which represented mean ± standard deviation from three independent repeats. Significance differences between treatments were analyzed by one-way ANOVA using Duncan’s multiple range tests (DMRT) implemented on agricolae package. Different letters on error bars represent significance differences at *p* ≤ 0.05.

To analyze the effect of *VdPAT1* gene on spore yield, prepared conidial suspension of each strain was inoculated in liquid CM medium. The conidial yield was determined using a hemocytometer under an optical microscope after 7 days of culture. It was found that the spore yield of Δ*VdPAT1*-*1* and Δ*VdPAT1*-*2* mutants was significantly lower than that of Vd592 and Δ*VdPAT1*-*C* strains (Figure 3A). To analyze the effect of *VdPAT1* gene on conidial germination rate, prepared conidial suspension of each strain was pipetted to the center of a microscope slide, incubated for 6 h in the dark, and observed under an optical microscope. It was found that the conidial germination rates of Δ*VdPAT1*-*1* and Δ*VdPAT1*-*2* mutants were significantly lower than that of Vd592 and Δ*VdPAT1*-*C* strains (Figure 3B). In contrast, there was no significance differences in conidial yield and conidial germination rates between Vd592 and Δ*VdPAT1*-*C* strain. These observations suggested that *VdPAT1* gene is involved in sporulation, and its deletion results in lower conidial yield and conidial germination rate of *V. dahliae*.

To investigate the effect of *VdPAT1* gene on mycelial growth, the Vd592, Δ*VdPAT1*-*1* and Δ*VdPAT1*-*2* mutants, and Δ*VdPAT1*-*C* strain were grown in liquid CM medium for 30 h and on PDA medium for 5 days, and then their mycelia were observed under an optical microscope. It was found that the Δ*VdPAT1*-*1* and Δ*VdPAT1*-*2* mutants produced significantly less mycelium compared to Vd592 and Δ*VdPAT1*-*C* strains on PDA medium (Figure 3C). We also found that the Δ*VdPAT1*-*1* and Δ*VdPAT1*-*2* mutants cultivated in both CM and PDA media produced impaired mycelium with abnormal branching, while Vd592 and Δ*VdPAT1*-*C* strain produced normal mycelium (Figure 3D). To analyze the effect of *VdPAT1* gene on mycelial penetration ability, the Vd592, Δ*VdPAT1*-*1* and Δ*VdPAT1*-*2* mutants, and Δ*VdPAT1*-*C* strain were cultivated on sterilized cellophane membranes which were overlaid on PDA media and incubated in the dark. After 7 days of culture, the cellophane membranes were removed (Figure 3E Top), and the PDA media were incubated in the dark for another 7 days. The result showed that the colony sizes of penetrating mycelium from Δ*VdPAT1*-*1* and Δ*VdPAT1*-*2* mutants were significantly smaller than that of Vd592 and Δ*VdPAT1*-*C* strain, suggesting that the Δ*VdPAT1*-*1* and Δ*VdPAT1*-*2* mutants have impaired mycelial penetrating ability (Figure 3E Bottom). Taken together, *VdPAT1* is required for mycelial growth and penetration of *V. dahliae*, its deletion resulted in less mycelial production, abnormal mycelial branching, and reduced penetration ability of *V. dahliae*.

### 2.4 *VdPAT1* is required for the pathogenicity of *Verticillium dahliae*

**Figure 4:**
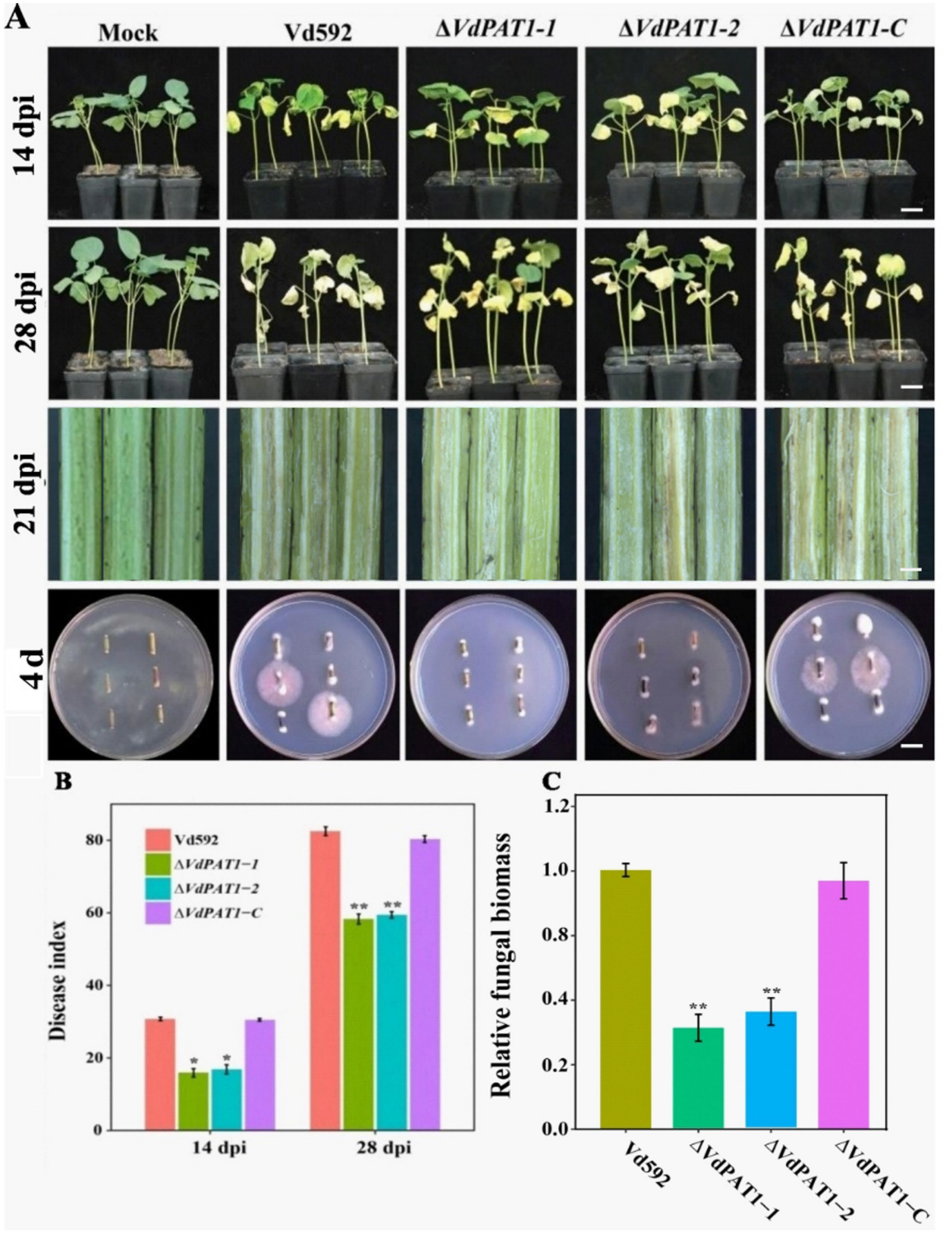
Pathogenicity assay of different *V. dahliae* strains. **(A)** The disease phenotypes of cotton plants infected with different strains at 14 dpi (days post infection) and 28 dpi. Scale bars represent 10 cm. Stem longitudinal sections of infected cotton plants at 21 dpi. Scale bars represent 1 cm. **(B)** Disease index of cotton plants infected with different strains at 14 dpi and 28 dpi. **(C)** The relative fungal biomass in cotton plants infected with different strains at 21 dpi. Data were statistically analyzed on R environment (version 4.3.2). The ggplot2 package was employed to generate bar plots which represented mean ± standard deviation from three independent repeats. Significance differences between treatments were analyzed by one-way ANOVA using Duncan’s multiple range tests (DMRT) implemented on agricolae package. Asterisks (*) and (**) on error bars represent significance differences at *p* ≤ 0.05 and *p* ≤ 0.01, respectively.

To investigate the effect *VdPAT1* on pathogenicity of *V. dahliae*, prepared spore suspension of Vd592, Δ*VdPAT1*-*1* and Δ*VdPAT1*-*2* mutants, and Δ*VdPAT1*-*C* strain was inoculated on a susceptible cotton variety by root injury method. At 14 and 28 dpi (days post inoculation), it was observed that the disease symptoms of the cotton seedlings infected with Δ*VdPAT1*-*1* and Δ*VdPAT1*-*2* mutants were obviously milder compared to the seedlings infected with Vd592 and Δ*VdPAT1*-*C* strains, displaying fewer yellowing and shedding of leaves and lighter browning of stem vascular bundles (Figure 4A). The disease index of cotton seedlings infected with Δ*VdPAT1*-*1* and Δ*VdPAT1*-*2* mutants were significantly lower than that of seedlings infected with Vd592 and Δ*VdPAT1*-*C* strains at both 14 and 28 dpi (Figure 4B). Fungal isolation assay showed that cotton seedlings infected with Δ*VdPAT1*-*1* and Δ*VdPAT1*-*2* mutants only contained a small amount of *V. dahliae* compared to seedlings infected with Vd592 and Δ*VdPAT1*-*C* strains (Figure 4A), which was confirmed by qRT-PCR detection (Figure 4C). However, there was no significant differences in disease index and fungal biomass between seedlings infected with Vd592 and Δ*VdPAT1*-*C* strains (Figures 4B,C). These results indicated that *VdPAT1* is required for the pathogenicity of *V. dahliae*, its deletion resulted in reduced fungal biomass in cotton stem and decreased pathogenicity of *V. dahliae*.

### 2.5 *VdPAT1* is required for the carbon, nitrogen and vitamin resources utilization of *Verticillium dahliae*

**Figure 5:**
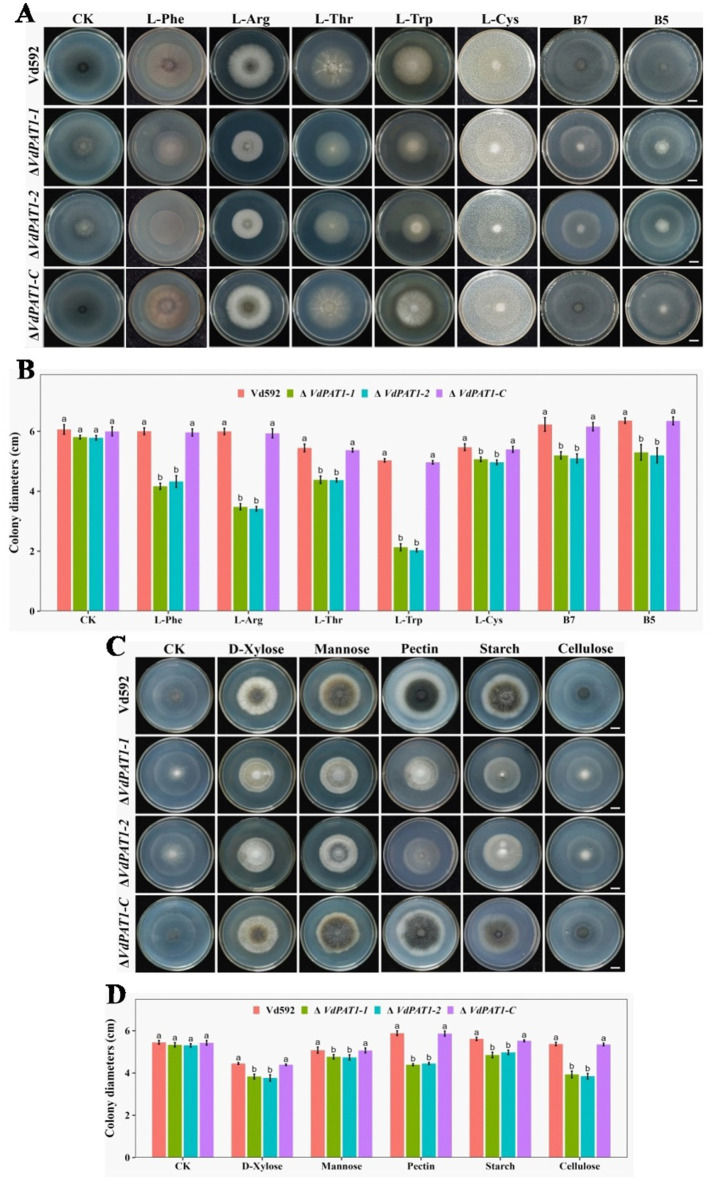
Colony morphology of different *V. dahliae* strains cultivated on different nitrogen, vitamin and carbon resources. **(A)** The colony morphology of different *V. dahliae* strains on Czapek medium plates supplemented with different amino acids (L-Phe, L-Arg, L-Thr, L-Trp or L-Cys) and vitamins (B7 or B5). Images were taken at 15 days post cultivation. CK represents different strains on Czapek medium without amino acids and vitamins. Scale bars represent 1 cm. **(B)** The colony diameters of different *V. dahliae* strains on Czapek medium supplemented with different amino acids and vitamins. **(C)** The colony morphology of different *V. dahliae* strains on Czapek medium plates supplemented with different carbon resources (D-xylose, mannose, pectin, starch or cellulose). Images were taken at 15 days post cultivation. CK represents different strains on Czapek medium without carbon resources. Scale bars represent 1 cm. **(D)** The colony diameters of different *V. dahliae* strains on Czapek medium plates supplemented with different carbon resources. Data were statistically analyzed on R environment (version 4.3.2). The ggplot2 package was employed to generate bar plots which represented mean ± standard deviation from three independent repeats. Significance differences between treatments were analyzed by one-way ANOVA using Duncan’s multiple range tests (DMRT) implemented on agricolae package. Different letters on error bars represents significance differences at *p* ≤ 0.05.

The decreased colony growth observed in *VdPAT1* deletion mutants was assumed to be a result of their reduced capacities to utilize a variety of nutrient resources, including sugars, amino acids and vitamins, etc. To verify this hypothesis, prepared spore suspension of Vd592, Δ*VdPAT1*-*1* and Δ*VdPAT1*-*2* mutants, and Δ*VdPAT1*-*C* strain was cultivated on Czapek medium supplemented with different amino acids (L-Phe, L-Arg, L-Thr, L-Trp or L-Cys), vitamins (B7 or B5) and sugars (D-xylose, mannose, pectin, starch or cellulose) (Figures 5A,C). The results showed that the colony diameters of Δ*VdPAT1*-*1* and Δ*VdPAT1*-*2* mutants were significantly smaller as compared to that of Vd592 and Δ*VdPAT1*-*C* on all Czapek media (Figures 5B,D), while there were no difference among different strains on Czapek medium without carbon, amino acid and vitamin resources (controls) (Figure 5). These results indicated that *VdPAT1* is required for the nutrition utilization of *V. dahliae*, its deletion resulted in reduced utilization capacities of carbon, nitrogen and vitamin resources of *V. dahliae*.

### 2.6 RNA-seq analysis of *VdPAT1* deletion mutant

**Figure 6:**
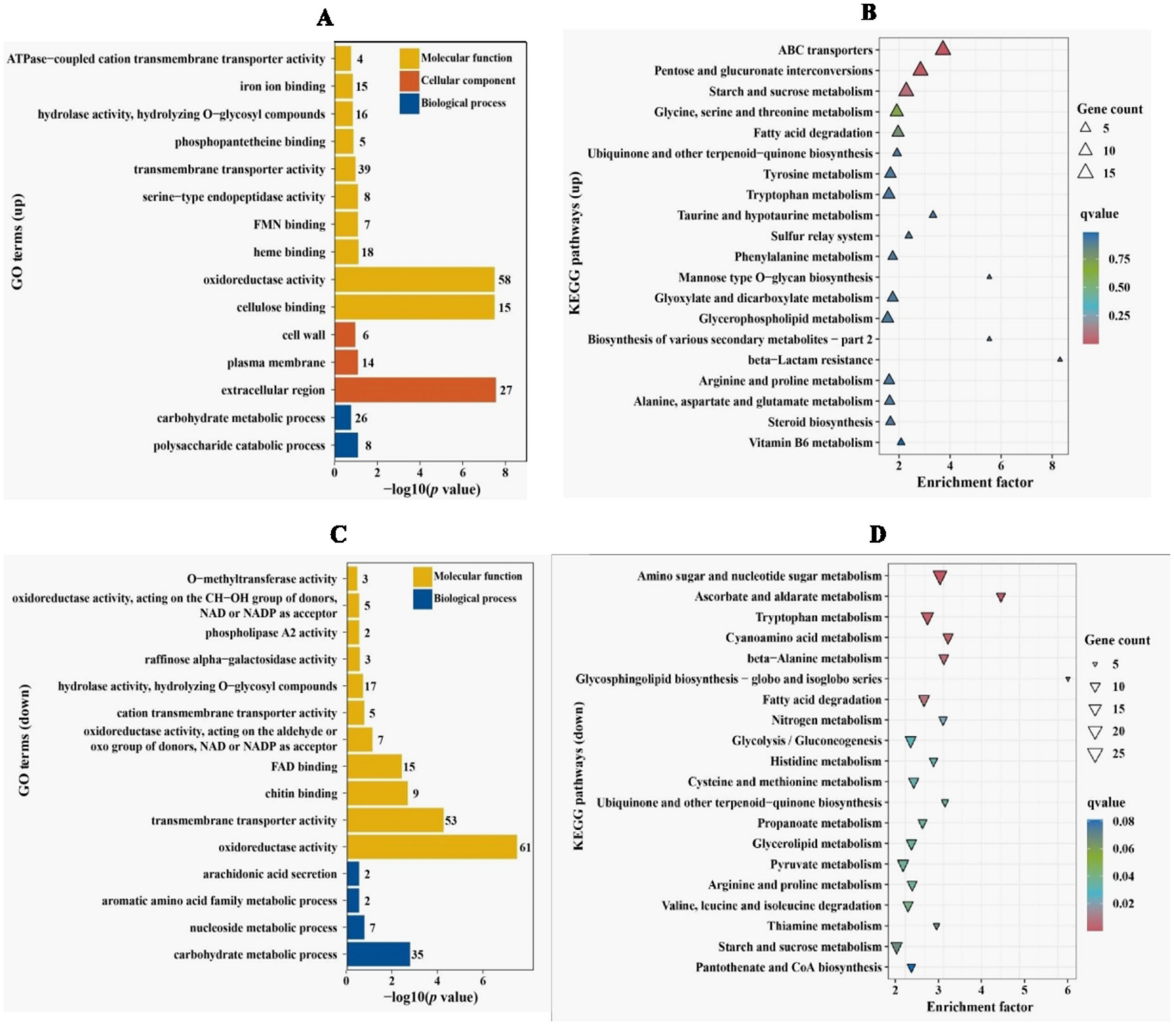
GO and KEGG enrichment analyses of differentially expressed genes. **(A)** GO enrichment analysis of up-regulated DEGs in *VdPAT1* deletion mutant. *X*-axis represents the −log_10_ (*p* value), and *Y*-axis represents the top 15 enriched GO terms. **(B)** KEGG pathways analysis of up-regulated DEGs in *VdPAT1* deletion mutant. *X*-axis represents the enrichment factor, and the *Y*-axis lists the top 20 enriched pathways. **(C)** GO enrichment analysis of down-regulated DEGs in *VdPAT1* deletion mutant. **(D)** KEGG pathways analysis of down-regulated DEGs in *VdPAT1* deletion mutant.

In order to clarify which gene expression changes lead to reduced penetration ability of *V. dahliae*, comparative transcriptome was applied to analyze differentially expressed genes (DEGs) between Vd592 and Δ*VdPAT1*-*1* mutant cultivated on sterilized cellophane membranes, which were overlaid on PDA medium. A total of 2,223 DEGs were identified, including 979 up-regulated and 1,244 down-regulated genes in Δ*VdPAT1*-*1* mutant. The RNA-seq results were validated to be reliable by qRT-PCR using 10 randomly picking DEGs (Supplementary Figures S2A,B). To further characterize the DEGs, we performed GO and KEGG analyses to categorize the up-and down-regulated DEGs. The top 15 most significantly enriched GO terms and the top 20 most significantly enriched KEGG pathways are presented in Figure 6. From the GO analysis, it was found that up-regulated DEGs were mainly enriched in MF (molecular function) category. Cellulose binding, oxidoreductase activity, and heme binding were the top three significantly enriched terms in the MF category. Extracellular region was the most significantly enriched term in CC (cellular component) category, and polysaccharide catabolic process was the most significantly enriched term in BP (biological process) category (Figure 6A). Down-regulated DEGs were also mainly enriched in MF category. Oxidoreductase activity, transmembrane transporter activity and chitin binding were the top three significantly enriched terms in the MF category. Carbohydrate metabolic process was the most significantly enriched term in BP category (Figure 6C). KEGG analysis found that ABC transporters, pentose and glucuronate interconversions, and starch and sucrose metabolism were the most significantly up-regulated pathways (Figure 6B). Amino sugar and nucleotide sugar metabolism, ascorbate and aldarate metabolism, and tryptophan metabolism were the most significantly down-regulated pathways (Figure 6D).

### 2.7 DEGs related to amino sugar and nucleotide sugar metabolism pathway

**Figure 7:**
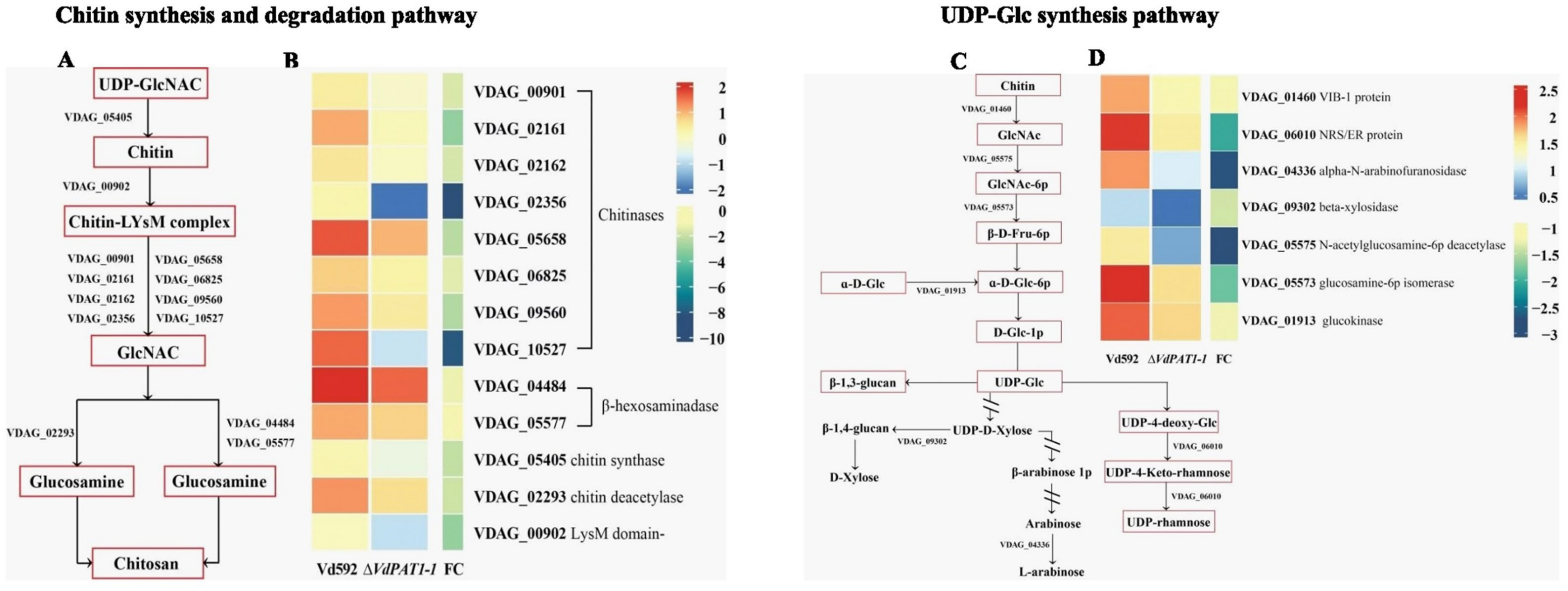
Heatmaps of DEGs related to chitin synthesis and degradation and UDP glucose synthesis. **(A)** A simple diagram showing the relationship of DEGs and chitin formation and decomposition. **(B)** A heatmap showing the expression level of DEGs related to chitin formation and decomposition. **(C)** A simple diagram showing the relationship of DEGs and UDP-glucose synthesis. **(D)** A heatmap showing the expression level of DEGs related to UDP-glucose synthesis. The heatmap was generated based on the FPKM value of genes provided by RNA-seq data.

It was notable that the most significantly enriched down-regulated pathway was amino sugar and nucleotide sugar metabolism pathway, which was related to chitin formation and UDP-glucose synthesis. A total of 25 DEGs were identified in this pathway. Of these DEGs, 13 were related to chitin formation and decomposition, consisting of 8 chitinases (VDAG_05658, VDAG_06825, VDAG_00901, VDAG_09560, VDAG_02162, VDAG_02161, VDAG_10527, and VDAG_02356), 1 chitin synthase (VDAG_05405), 1 chitin deacetylase (VDAG_02293), 2 beta-hexosaminadase (VDAG_05577 and VDAG_04484) and 1 LysM domain-containing protein (VDAG_00902) (Figures 7A,B) genes. Seven DEGs were found to be related to UDP-glucose synthesis, including VIB-1 protein (VDAG_01460), glucokinase (VDAG_01913), NRS/ER (VDAG_06010), β-xylosidase (VDAG_09302), N-acetylglucosamine-6-phosphate-deacetylase (VDAG_05575), glucosamine-6-phosphate deaminase (VDAG_05573) and α-L-arabinofuranosidase (VDAG_04336) genes (Figures 7C,D).

**Figure 8:**
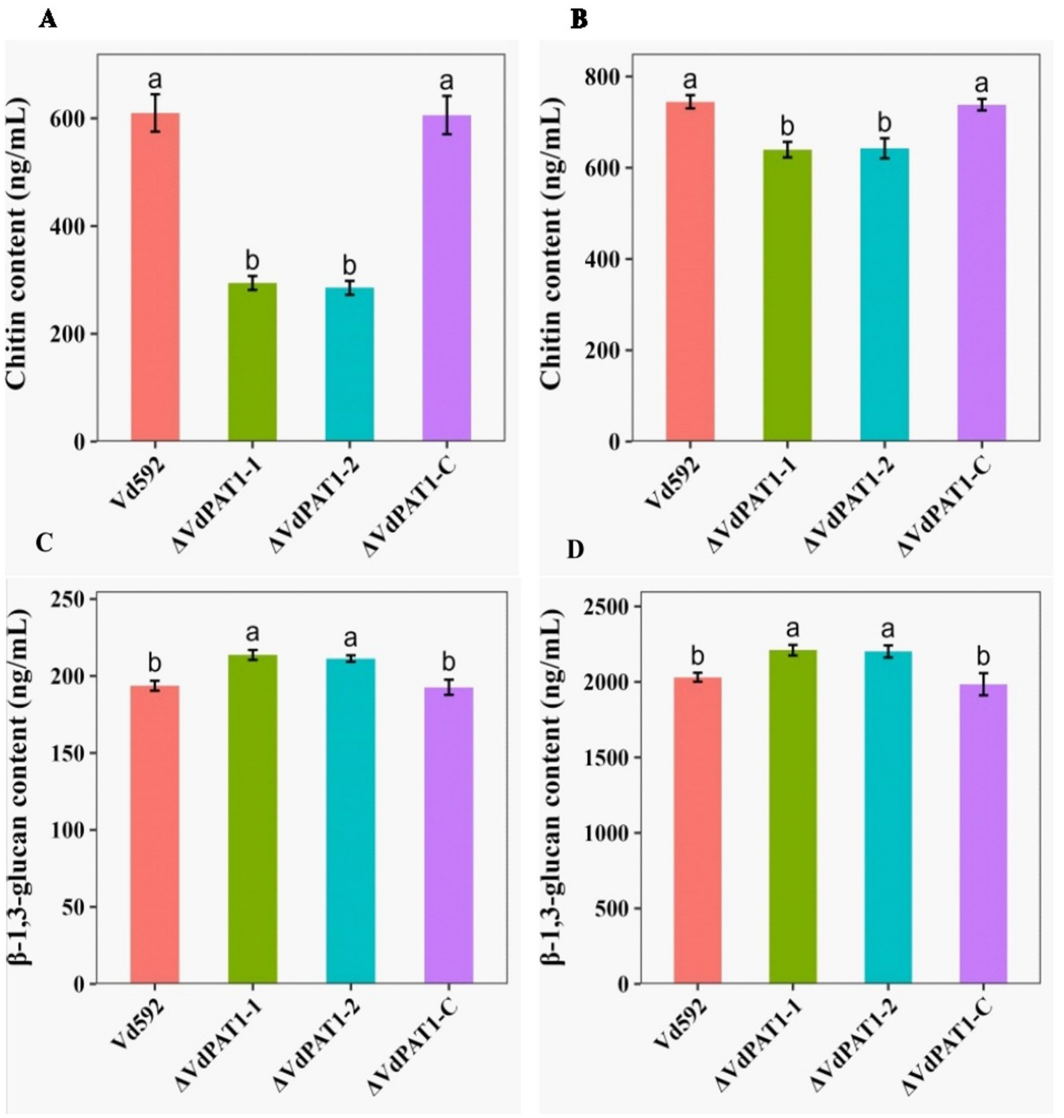
Chitin and β-1,3-glucan content of different *V. dahliae* strains. **(A)** Chitin content of different *V. dahliae* strains by using the same samples as RNA-seq. **(B)** Chitin content of different *V. dahliae* strains by using the samples cultured in liquid CM medium. **(C)** β-1,3-glucan content of different *V. dahliae* strains by using the same samples as RNA-seq. **(D)** β-1,3-glucan content of different *V. dahliae* strains by using the samples cultured in liquid CM medium. The abbreviations (ng/mL) on *Y*-axis means nanogram per milliliter of the sample. Data were statistically analyzed on R environment (version 4.3.2). The ggplot2 package was employed to generate bar plots which represented mean ± standard deviation from three independent repeats. Significance differences between treatments were analyzed by one-way ANOVA using Duncan’s multiple range tests (DMRT) implemented on agricolae package. Different letters on error bars represents significance differences at *p* ≤ 0.05.

Chitin is an important structural component of fungal cell wall, but it is absent from plants and vertebrates (Maertens and Boogaerts, 2000). UDP-glucose is a precursor of β-1,3-glucan and β-1,6-glucan. β-1,3-glucan can link to chitin constituting the backbone of the fungal cell wall matrix (Orlean, 2012). The ratio change of β-1,3-glucan and chitin content has been found to lead to the abnormal morphogenesis of fungal cells (Cantu et al., 2009). The *V. dahliae* samples same as those used in RNA-seq were therefore used for the content determination of chitin and β-1,3-glucan. The results showed that the chitin content of the Δ*VdPAT1*-*1* and Δ*VdPAT1*-*2* mutants were 29.4 ng/mL and 28.5 ng/mL, respectively, significantly lower than that of Vd592 (61 1 ng/mL) and Δ*VdPAT1*-C (60.6 ng/mL) strains (Figure 8A). The β-1,3-glucan content of the Δ*VdPAT1*-*1* and Δ*VdPAT1*-*2* mutants were 21.4 ng/mL and 21.1 ng/mL, respectively, significantly higher than that of Vd592 (19.4 ng/mL) and Δ*VdPAT1*-*C* strains (19.3 ng/mL) (Figure 8C). The fungal samples cultured in liquid CM medium for 5 days were also used for chitin and β-1,3-glucan content determination, obtaining similar results as that observed in the fungal samples grown on cellophane membrane (Figures 8B,D). The decreased chitin content and increased β-1,3-glucan content in *VdPAT1* deletion mutants may lead to abnormal morphogenesis of *V. dahliae* cells, such as abnormal branching of hyphae, resulting in the reduced penetration ability of *V. dahliae*.

### 2.8 *VdPAT1* is required for cell wall integrity and stress resistance of *Verticillium dahliae*

**Figure 9:**
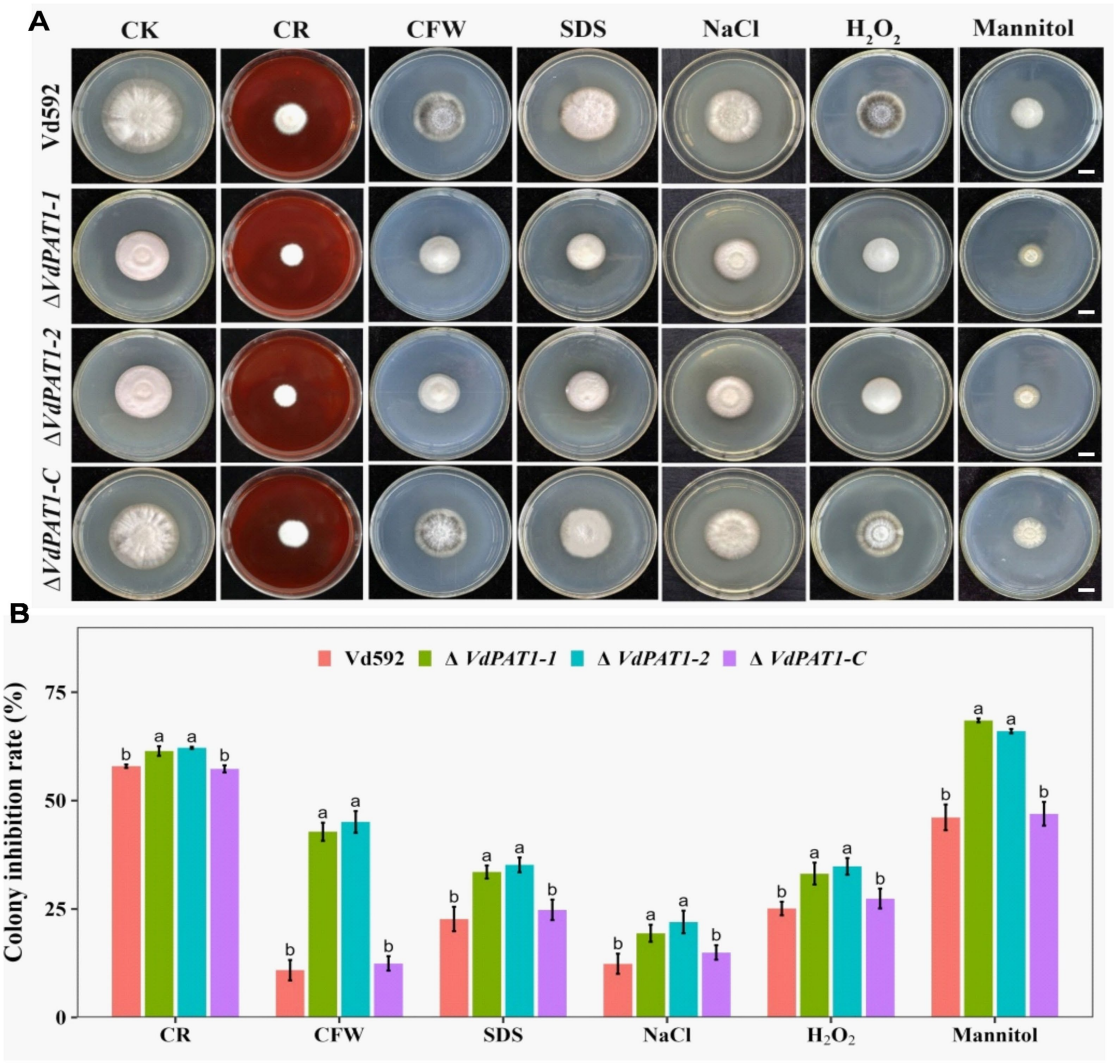
Colony morphology of different *V. dahliae* strains cultivated on cell wall perturbing agents and other external stress agents. **(A)** The colony morphology of different *V. dahliae* strains on CM medium plates supplemented with cell wall perturbing agents (Congo red, CFW or SDS) and other external stresses agents (NaCl, H_2_O_2_ or mannitol). The CK represents different strains cultivated on CM medium without stress agents. Scale bars represent 1 cm. Images were taken at 15 days post cultivation. **(B)** The colony inhibition rates of different *V. dahliae* strains on CM medium plates supplemented with cell wall perturbing agents and other external stresses agents. Data were statistically analyzed on R environment (version 4.3.2). The ggplot2 package was employed to generate bar plots which represented mean ± standard deviation from three independent repeats. Significance differences between treatments were analyzed by one-way ANOVA using Duncan’s multiple range tests (DMRT) implemented on agricolae package. Different letters on error bars represents significance differences at *p* ≤ 0.05.

Chitin maintains cellular integrity and offers resistance to environmental stress (Qin et al., 2022). The decreased chitin content in Δ*VdPAT1* mutant made us speculated that the cell wall integrity and stress resistance of Δ*VdPAT1* mutant strain may be affected. To verify this hypothesis, we compared the growth inhibition rates of different *V. dahliae* strains on CM medium containing the cell wall perturbing agents CFW (calcoflour white), CR (Congo red), or SDS (sodium dodecycl sulfate). As presented in Figure 9A, the colony growth inhibition rates of Δ*VdPAT1*-*1* and Δ*VdPAT1*-*2* mutants were significantly higher in CFW, CR and SDS media as compared to that of Vd592 and complementary strain (Figure 9B), suggesting that the cell wall integrity of *VdPAT1* deletion mutants were impaired, resulting in *V. dahliae* being more sensitive to cell wall integrity. We also examined the effect of more external stresses (H_2_O_2_, mannitol and NaCl) on the *VdPAT1* deletion mutants, and obtained similar results (Figures 9A,B) to those of cell wall perturbing agents, suggesting that *VdPAT1* deletion mutants are more sensitive to external stresses, possibly by impairing the fungal cell wall integrity. These observations suggested that *VdPAT1* is involved in cell wall integrity maintenance and stress resilience of *V. dahliae*.

### 2.9 Other DEGs

Our RNA-seq data also identified 22 down-regulated DEGs in the Δ*VdPAT1* mutant that participate in pathogen-host interactions (PHI) (Table 1) (Supplementary Figure S3A). Of the 22 HPI genes, 8 (VDAG_06010, VDAG_04701, VDAG_02630, VDAG_02354, VDAG_10392, VDAG_09950, VDAG_00902, and VDAG_08100) have been reported to be related to the pathogenicity of *V. dahliae*, other unreported genes were found to match some reported HPI genes from other fungi (Table 1). There is redundancy in gene family members, so if the function of one gene is knocked out, it may be compensated by other members (Khanppnavar et al., 2019). It was notable that the Δ*VdPAT1*-*1* and Δ*VdPAT1*-*2* mutants grown on Czapek medium containing vitamin B5 cannot restore normal growth as wild-type, suggesting that no other pantothenate transporter genes can compensate its function. A total of 10 pantothenate transporter genes were identified in *V. dahliae*, while only one (VDAG_09374) was found to be differentially expressed between *VdPAT1*-*1* mutant and wild-type (Supplementary Figure S3B). These results indicated that VDAG_09374 cannot compensate the function of *VdPAT1*, although it showed more than 5-fold up-regulation in Δ*VdPAT1*-*1* mutant.

**Table 1:** The list of down-regulated PHI genes in the Δ*VdPAT1-1* mutant based on RNA-seq data.

PHI genes	Related genes	Pathogen species	Mutant phenotypic characteristic	References
VDAG_05297	*ABA4*	*M. oryzae*	Reduced virulence	Spence et al. (2015)
VDAG_05884	*MoGLN2*	*M. oryzae*	Loss of pathogenicity	Aron et al. (2021)
VDAG_06010	*VdNRS/ER*	*V. dahliae*	Loss of pathogenicity	Santhanam et al. (2017)
VDAG_06751	*MoPRX1*	*M. oryzae*	Reduced virulence	Mir et al. (2015)
VDAG_05175	*β-tubulin*	*V. inaequalis*	Resistance to chemical	Koenraadt et al. (1992)
VDAG_04701	*NLP1*	*V. dahliae*	Reduce virulence	Song and Thomma (2018)
VDAG_04571	*gta1*	*F. graminearum*	Reduced virulence	Bönnighausen et al. (2015)
VDAG_03272	*MgSAM1*	*M. oryzae*	Reduced virulence	Saint-Macary et al. (2015)
VDAG_02853	*MoTPS2*	*M. oryzae*	Reduced virulence	Chen et al. (2021)
VDAG_02630	*VdSOD1*	*V. dahliae*	Reduced virulence	Tian et al. (2021)
VDAG_02409	*Smt3*	*M. oryzae*	Reduced virulence	Lim et al. (2018)
VDAG_02354	*VdHog1*	*V. dahliae*	Reduced virulence	Wang et al. (2016)
VDAG_10392	*ACC deaminase*	*V. dahliae*	Reduced virulence	Tsolakidou et al. (2019)
VDAG_09950	*HiC-15*	*V. dahliae*	Reduced virulence	Zhang et al. (2016)
VDAG_01774	*MIs1*	*S. nodorum*	Loss of pathogenicity	Solomon et al. (2004)
VDAG_09046	*Ss-pth2*	*S. scleortiorum*	Reduced virulence	Liberti et al. (2013)
VDAG_08981	*AGL1*	*M. oryzae*	Reduced virulence	Badaruddin et al. (2013)
VDAG_00902	*Vd4LysM*	*V. dahliae*	Effector (a virulence determinant)	Kombrink et al. (2017)
VDAG_08100	*VdSSEP1*	*V. dahliae*	Reduced virulence	Han et al. (2019)
VDAG_07814	*Fgsah1*	*F. graminearum*	Reduced virulence	Shi et al. (2021)
VDAG_07784	*MoCDIP4*	*M. oryzae*	Effector (a virulence determinant)	Chen et al. (2013)
VDAG_00364	*UBI1*	*C. neoformans*	Reduced virulence	Zhao et al. (2020)

## 3 Discussion

### 3.1 *VdPAT1*, a member of the MFS superfamily, is required for growth, development and pathogenicity of *Verticillium dahliae*

In this study, a pantothenate transporter gene, *VdPAT1*, was selected for functional characterization. *VdPAT1* encodes a protein with 489aa residues and contains an MFS domain and 12 TMDs, suggesting that it belongs to MFS superfamily, which is one of the largest families of membrane carrier proteins (Reddy et al., 2012). The members of this superfamily typically contain 400 to 600aa residues and 12 putative transmembrane domains (TMDs). These domains enable transportation of a variety of molecules, including sugars, drugs, amino acids and vitamins (Bagchi et al., 2020; Chen et al., 2008; Yen et al., 2010). According to Transporter Classification Database (TCDB; http://www.tcd.org), the MFS superfamily is categorized into 17 subfamilies based on evolutionary relationships and protein roles (Saier, 1998). Among these, the ACS subfamily is one of the largest and most diverse, comprising of 40 members from bacteria, animals and *Saccharomyces cerevisiae* (Pao et al., 1998). The members of this family are recognized by an ACS consensus motif sequence in their fourth transmembrane domain which harbors 7 residues (GEPWPER) (Pao et al., 1998). Multiple sequence alignment found that *VdPAT1* harbored the ACS consensus motif sequence, suggesting that it belongs to ACS subfamily.

Pantothenate is essential for fungal growth and development (Ipcho et al., 2012). Pantothenate transporter genes are important for the uptake of exogenous pantothenate in living organisms (Meir and Osherov, 2018). Several pantothenate transporter genes have been identified from fungi, and their functions in fungal growth, development, nutrient uptake and pathogenesis process have been studied (Stolz and Sauer, 1999). However, no relevant reports have been found in *V. dahliae*. In this study, we generated *VdPAT1* deletion mutants and complementary strains and used them in functional characterization. The *VdPAT1* deletion mutants displayed reduced colony growth, spore yield and germination rate, leading to decreased pathogenicity, consistent with the previous reports (Dietl et al., 2018; Stolz and Sauer, 1999; Stolz et al., 2004; White et al., 2001; Yang et al., 2023).

### 3.2 Down-regulation of amino sugar and nucleotide sugar metabolism pathway leads to the reduced penetration ability of *Verticillium dahliae*

The fungal cell wall is made up of complex polysaccharides including β-1,3-glucan synthesized from UDP-Glc and chitin, a β-1, 4-linked polymer of N-acetyl glucosamine (Daran et al., 1995; Lenardon et al., 2010). Disrupting genes necessary for cell wall synthesis affects growth, reproduction and pathogenicity of the fungus (Gow et al., 2017). For instance, deletion of a chitin synthase gene *CHS1* in *Magnaporthe oryzae* was found to disrupt appressorium formation and hinder appressorium penetration (Lu et al., 2023). Deletion of four chitin synthase genes (*VdCHS1*, *VdCHS4*, *VdCHS6*, and *VdCHS7*) in *V. dahliae* was found to impair the *in vitro* and *in vivo* penetration abilities of *V. dahliae* (Qin et al., 2022). In *Aspergillus niger*, double deletion of a chitinase oligosaccharide gene *CfcI* and a endochitinase gene *CtcB* was found to interfere with cell walls during sporulation (Munster et al., 2013). Deletion of a chitin deacetylase gene in *V. dahliae* was found to reduce the fungal virulence (Gao et al., 2019). Deletion of a β-1,3-glucan synthesis gene *GLS1* in *Collectotrichum graminicola* was found to impair hyphal cell wall, affecting appressorium function (Oliveira-Garcia and Deising, 2013). Deletion of a nucleotide-rhamnose synthase/epimerase reductase (*VdNRS/ER*) gene in *V. dahliae* was found to inhibit UDP-rhamnose synthesis, interfering with fungal cell wall and pathogenicity (Santhanam et al., 2017).

In this study, *VdPAT1* deletion resulted in impaired mycelial growth, abnormal mycelial branching and reduced penetration ability. RNA-seq analysis between Vd592 and Δ*VdPAT1* was conducted to explore the reason why the mutants have the reduced penetration ability. It was notable that the most significantly enriched pathways among the down-regulated pathways was amino sugar and nucleotide sugar metabolism pathways, which are related to chitin synthesis and degradation as well as UDP-Glc synthesis. The ratio change of β-1,3-glucan and chitin content has been found to lead to the abnormal morphogenesis of fungal cells (Cantu et al., 2009; Geoghegan et al., 2017; Samalova et al., 2016). Here, the *VdPAT1* deletion affected the expression of several genes related to chitin synthesis and degradation as well as UDP-Glc synthesis, and changed the ratio of β-1,3-glucan and chitin content, which were likely responsible for the impaired mycelial growth, abnormal mycelial branching and reduced penetration ability.

### 3.3 Down-regulation of amino sugar and nucleotide sugar metabolism pathway is associated with the increased environmental sensitivity of *Verticillium dahliae*

Previous studies have reported that cell wall integrity maintenance is preserved across different species of fungi and is vital for fungi to resist stress and synthesize cell wall (Levin, 2011). Changes in the expression of genes related to cell wall synthesis affect the environmental sensitivity of fungi. For example, disrupting chitin synthase genes (*CHS*) in *V. dahliae* has been found to affect the fungal environmental sensitivity. The *Vdchs5* mutant displayed hypersensitivity when cultured on PDA containing NaCl and sorbitol. The *Vdchs1* and *Vdchs4* mutants were sensitive only to NaCl, while the *Vdchs3* and *Vdchs6* mutants were sensitive to sorbitol. In addition, the *Vdchs3* and *Vdchs5* mutants displayed sensitivity to perturbing agents such as CR and CFW (Qin et al., 2022). Knockout of a UTP-glucose-1-phosphate uridylyltransferase gene (*VdUGP*) in *V. dahliae* resulted in the fungi to be sensitive to SDS and high concentrations of NaCl (Deng et al., 2020).

Chitin, as a key component of the fungal cell wall, protects the fungal cell against external stresses and responds to changes in environmental conditions (Talbot et al., 1996). Chitin maintains the cellular integrity and resistance to environmental stress (Qin et al., 2022). Changes in the composition of the fungal cell wall can cause changes in the ability of fungi to resist external stresses (Ma et al., 2017). In this study, *VdPAT1* deletion mutants were more sensitive to external stresses, including CR, CFW, SDS, NaCl, H_2_O_2_ and mannitol. The *VdPAT1* deletion resulted in the down-regulation of several genes related to chitin synthesis and degradation as well as UDP-glucose synthesis and decreased chitin content, which is likely to be the reason for the increased sensitivity of *VdPAT1* mutant to environmental stresses. Deletion or inhibition of genes responsible for pantothenate utilization has been found to significantly impair the fungal resistance capability to environmental stresses (Chiu et al., 2019; Gihaz et al., 2022; Lussier et al., 1997), but the underlying molecular metabolism is still unknown. Our RNA-seq results provide the molecular evidence to explain the increased environmental sensitivity of fungi after knocking out the gene responsible for pantothenate utilization.

Pantothenate is an integral substrate in the production of coenzyme (CoA), a molecule essential for energy production and lipid metabolism in organisms (Leonardi and Jackowski, 2007; Spry et al., 2008). Disruption of pantothenate-coenzyme-A-(CoA)-Acetyl-(AcCoA) (PCA) pathway in *S. cerevisiae* was found to cause a significant change in the fungus susceptibility to a variety of xenobiotics, such as heavy metals. The inability of fungus to carryout detoxification process resulted in vacuolar organelle damage leading to mitochondrial abnormalities due to increased reactive oxygen species (ROS) levels (Choi et al., 2024). Pantothenate stressors, like pantothenamide alphaAM (αpanAM), were found to hinder the growth of *S. cerevisiae* and interfered with pantothenate utilization and coenzyme (CoA) biosynthesis (Chiu et al., 2017). In other pathogenic microorganisms, such as bacteria *Staphylococcus aureus*, inhibitors like pantothenamide were found to restrict the growth of the bacteria by preventing pantothenate phosphorylation reaction catalyzed by pantothenate kinase (PanK) enzyme (Leonardi et al., 2005).

**Figure 10:**
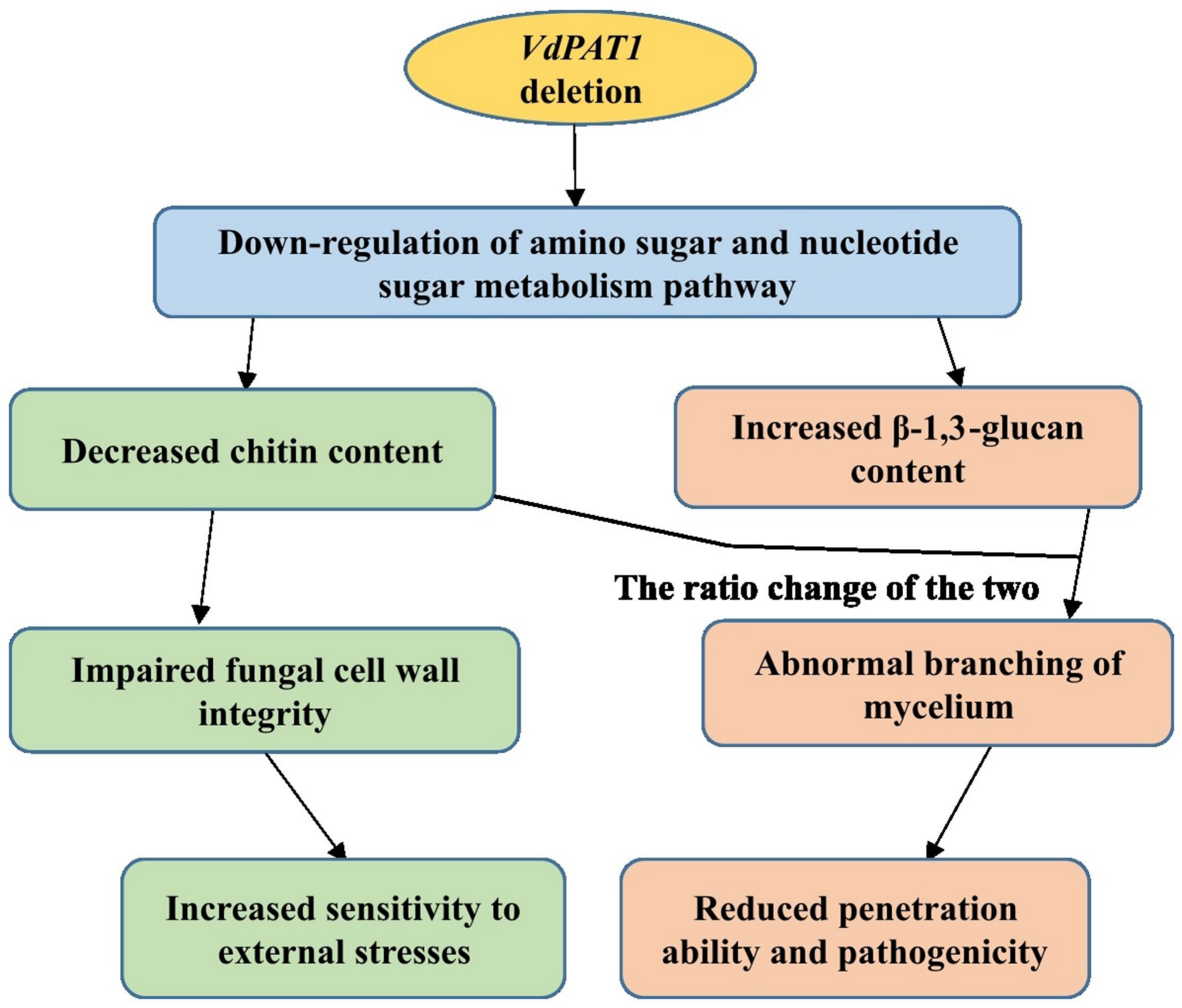
A working model for the role of *VdPAT1* in the mycelial growth, stress resistance and pathogenicity of *V. dahliae*.

In conclusion, the pantothenate transporter *VdPAT1* is required for growth, development, resistance to external stresses, mycelial penetration and pathogenicity of *V. dahliae*. *VdPAT1* deletion resulted in reduced colony growth, spore yield and germination rate, abnormal mycelial branching, decreased ability of mycelial penetration and utilization of nutrients (carbon, amino acids and vitamin), and a lower pathogenicity of *V. dahliae*. Comparative transcriptome analysis of wild-type and *VdPAT1* deletion mutant cultivated on sterilized carboxymethyl cellophane membranes found that the amino sugar and nucleotide sugar metabolism pathway, which was related to chitin synthesis and degradation as well as UDP-glucose synthesis, was significantly down-regulated in *VdPAT1* deletion mutant. The chitin content in *VdPAT1* deletion mutants was significantly lower, while β-1,3-glucan content was higher than that of wild-type. The ratio change of chitin and β-1,3-glucan content in *VdPAT1* deletion mutants might lead to abnormal branching of mycelium, resulting in the reduced penetration ability of *V. dahliae*. The decreased chitin content in *VdPAT1* mutants impaired the fungal cell wall integrity, leading to their increased sensitivity to external stresses (Figure 10).

## 4 Materials and methods

### 4.1 Plasmids, fungal strain and plant materials

The pGKO_2_-Gate and pSULPH-mut-RG#PB vectors used in this study were kindly donated by Dr. Zhaosheng Kong, Institute of Microbiology, and Chinese Academy of Sciences. *V. dahliae* Vd592, a highly aggressive strain, is preserved in PDA (potato dextrose agar) medium containing 60% glycerine and stored at −80°C in our laboratory. The strain was cultivated in liquid CM medium (complete medium) at 25°C with shaking (200 rpm) for 5 days. The fungal suspension was filtered with 8 layers of gauze to obtain conidia. The collected conidia were diluted with sterile water to a concentration of 1 × 10^7^ CFU/mL for the following experiments. Upland cotton variety “Xinzulao 7” susceptible to *V. dahliae* was used in this study. This variety was donated by the Cotton Research Institute of Shihezi University. Cotton seeds were first pre-germinated in a growth chamber at 28°C for 36 h. The germinated seeds were grown in pots filled with nutrient soil and vermiculite (3:1) ratio, with one seed planted in each pot. These pots were put in a greenhouse with a photoperiod of 16 h light/8 h dark and a relative humidity of 60% at 25°C for a duration of 4 weeks.

### 4.2 Bioinformatics analysis

The protein sequences, GFF3, CDS, and genome reference of *V. dahliae* were downloaded from online platform.^1^ Using a single *Saccharomyces cerevisiae Tna1* gene as a query sequence, 10 pantothenate transporter genes were identified from the GFF3 file of *V. dahliae* using online TBtools software^2^ and were validated by BlastP in the NCBI database.^3^ The domain structure was identified using SMART tool software,^4^ which integrates HMMER3 tool with Pfam database and was visualized using DOG1.0 illustrator.^5^ Deep TMHMM software tool was used to predict transmembrane structural domains (TMD).^6^ Phylogenetic tree was constructed by MEGA 11.0 using the neighbor-joining method and was visualized by Interactive Tree of Life tool.^7^ Multiple sequence alignment was visualized using PFAAT software version 2.0. The R package of pheatmap was employed to generate the heatmaps.

### 4.3 Generation and confirmation of *VdPAT1* deletion mutants and complementary strain

The DNA of *V. dahliae* was extracted using a fungal DNA Kit (Omega Inc., United States). The total RNA of *V. dahliae* was extracted using a fungal RNA kit (Omega Inc., United States). The RNA was reversely transcribed to cDNA using an EasyScript One-Step gDNA Removal and cDNA Synthesis Super Mix EasyScript kit following the manufacturer’s instructions.

For deletion vector construction, a 1,000 bp long target fragment of *VdPAT1* was replaced by a 1,908 bp long fragment of a hygromycin resistance gene (*HPH*), which was amplified from T + *HPH* plasmid with *HPH*-F/R primers (Supplementary Table S1). Upstream (1,000 bp) and downstream (1,000 bp) flanking fragments of the target fragment were amplified from Vd592 genomic DNA with paired primers *VdPAT1*-flank-5F/5R and *VdPAT1*-flank-3F/3R (Supplementary Table S1). The three amplified fragments were then recombined into pGKO2-Gate knockout vector using a ClonExpress II one-step cloning kit (Vazyme Biotech Co., Ltd., Nanjing, China) following the manufacturer’s instructions. The recombinant vector was transformed into Vd592 using a PEG-mediated transformation method (Wang et al., 2012). The transformants were screened on PDA medium containing suitable antibiotics and confirmed by PCR and qRT-PCR by using primers: Trial 1-*VdPAT1*-F1/R1, Trial 2-*VdPAT1*-F2/R2, and Trial 3-*VdPAT1*-F3/R3 (Supplementary Table S1). For complementary vector construction, a 4,700 bp long fragment containing promoter, coding region and terminator sequences of *VdPAT1* was amplified from Vd592 genomic DNA with promoter-F/*VdPAT1*-R primers (Supplementary Table S1). The amplified fragment was combined into the pSULPH-mut-PG#PB vector using the ClonExpress II one-step cloning kit. The recombinant vector was transformed into *VdPAT1* deletion mutant using the PEG-mediated transformation method. The transformants were confirmed by PCR and qRT-PCR. The qRT-PCR was performed using SYBR premix EX Taq (TakaRa) on a LightCycler 480 system II (Roche, United States) instrument. The relative expression ratio of each gene was calculated from the cycle threshold (Ct) values as proposed by Livak and Schmittgen (2001). *V. dahliae* β-tubulin gene (VDAG_10074) was used as an endogenous control.

### 4.4 Investigation of fungal morphological characteristics

For determination of colony growth diameter, 10 μL of conidial suspension (1 × 10^7^ CFU/mL) of each strain was dropped onto the center of petri dishes containing PDA (Potato Dextrose Agar), CM (Complete Medium) and Czapek (Czapek Dox Agar) media, respectively, and incubated at 25°C. PDA is a basic medium, which is well balanced in terms of nutritional requirements for the growth and development of microorganisms and is commonly used for the fungal cultivation. CM medium contains a variety of vitamins and trace mineral elements that are useful for fungal growth (Chen et al., 2023). Czapek is a nutrient medium used for general cultivation of fungi and it contains sucrose as the main carbon source, sodium nitrate as a nitrogen source and other inorganic salts (Szatmari et al., 2015). The colony diameter of each strain was measured every 3 days and photographed at 15 days post incubation. Three independent repeats were performed.

For observation of hyphal morphology, 10 μL of conidial suspension (1 × 10^7^ CFU/mL) of each strain was spotted on the sides of a 5 mm PDA block which was overlaid on a microscope slide placed on a sterile moist filter paper in a petri dish, and then incubated in the dark for 3 days. The hyphal morphology of each strain was observed and photographed using a stereo-microscope (Zeiss, Germany). Three independent repeats were performed.

For determination of conidial yield, 50 μL of conidial suspension (1 × 10^7^ CFU/mL) of each strain was dropped into 50 mL liquid CM medium and incubated at 25°C with shaking (200 rpm/min) in the dark. The conidial number of each strain were determined using a hemocytometer under an optical microscope for 7 consecutive days. For determination of conidial germination rate, 10 μL of conidial suspension (1 × 10^7^ CFU/mL) of each strain was dropped onto the center of a microscope slide which was overlaid on sterilized moisture filter paper and incubated in the dark for 6 h. conidial germination rate was determined under an optical microscope. Three independent repeats were performed.

For mycelial penetration ability determination assay, 10 μL conidial suspension (1 × 10^7^ CFU/mL) of each strain was dropped onto sterilized carboxymethyl cellulose membrane overlaid on PDA medium and incubated for 7 days in the dark. After removing the carboxymethyl cellulose membrane, the PDA medium was incubated for another 7 days, and the colony of each strain was observed and photographed as previously described (Tian et al., 2021). Three independent repeats were performed.

### 4.5 Stress response assays

To test the cell wall integrity of each strain, 10 μL of conidial suspension (1 × 10^7^ CFU/mL) of each strain was dropped onto the center of CM medium containing cell wall inhibitor agents Congo red (50 μg/mL), CFW (10 μg/mL), and SDS (50 μg/mL of 0.01%) (Yu et al., 2019), respectively. The CM medium without cell wall inhibitor agents was used as control. The colony width of each strain was measured and photographed at 12 days post incubation. Three independent repeats were performed.

To test the response of each strain to osmotic stress, 10 μL of conidial suspension (1 × 10^7^ CFU/mL) was dropped onto the center of CM medium containing, NaCl (0.4 M), H_2_O_2_ (0.03 M), and mannitol (0.2 M), respectively. The CM medium without osmotic agents was used as control. The colony width of each strain was measured and photographed at 12 days post incubation. To determine the colony inhibition rate, the formula proposed by Albuquerque et al. (2006) was employed: inhibition rate % = [(control growth diameter − treatment growth diameter)/control growth diameter] × 100. Three independent repeats were performed.

### 4.6 Carbon, nitrogen and vitamin utilization assays

To test the carbon source utilization capacity of each strain, 10 μL of conidial suspension (1 × 10^7^ CFU/mL) was dropped onto the center of Czapek medium containing D-xylose (1%), mannose (1%), pectin (1%), starch (1%), and cellulose (1%), respectively. The Czapek medium without carbon resources was used as control. The colony diameter of each strain was measured and photographed at 15 days post incubation (Qi et al., 2016). Three independent repeats were performed.

To test the nitrogen utilization capacity of each strain, 10 μL of conidial suspension (1 × 10^7^ CFU/mL) was dropped onto the center of Czapek medium containing L-phenylalanine (1%), L-arginine (1%), L-threonine (1%), L-tryptophan (1%), and L-cysteine (1%), respectively. The Czapek medium without nitrogen resources was used as control. The colony width of each strain was measured and photographed at 15 days post incubation (Wong et al., 2008). Three independent repeats were performed.

To test the vitamin uptake capacity of each strain, 10 μL of conidial suspension (1 × 10^7^ CFU/mL) was dropped onto the center of Czapek medium containing B7 (0.1 mg/L) and B5 (0.1 mg/L), respectively. The colony width of each strain was measured and photographed at 15 days post incubation (Burkholder and Moyer, 1943). Three independent repeats were performed.

### 4.7 Pathogenicity assay

In brief, when the cotton seedlings reached two true leaf stage, 20 mL of conidial suspension (1 × 10^7^ CFU/mL) was injected into the bottom of each pot to infect cotton roots. Cotton plants treated with double distilled sterile water (ddH_2_O) were used as control (mock). Three independent replicates were performed, each consisting of 10 seedlings. The disease symptoms were observed and photographed, and the disease index (DI) were evaluated at 14 and 28 dpi (days post infection). To determine the disease index, disease grades were first classified into four grades ranging from (0 to 4) based on the wilting % on the leaves (Wang et al., 2020). The disease index for each treatment was then calculated using the formula previously described (Wang et al., 2020; Zhang et al., 2017) where disease index (DI) = [(∑ number of diseased plants × corresponding disease grade)/(total number of plants sampled × 4)] × 100. The infected stems were longitudinally cut using sterile scalpel at 21 dpi, and the vascular discoloration was observed and photographed. For the fungal recovery assay, infected stems were collected at 21 dpi, sterilized, and then cut into segments of 2–3 cm. The stem segments were cultivated on PDA medium and incubated at 25°C in the dark. The fungal mycelia generated from the stem segments were observed and photographed after 4 days of incubation. For fungal biomass detection, the infected stems were collected at 21 dpi and used for genomic DNA extraction. The qRT-PCR was performed with specific primers ITS1-F and ST-Ve1-R. The cotton *GhUBQ7* gene was used as the endogenous control (Supplementary Table S1).

### 4.8 RNA-sequencing and analysis of differentially expressed genes

For RNA-seq, 10 μL of the conidial suspension of Vd592 and *VdPT1* deletion mutant (Δ*VdPT1-1*) (1 × 10^7^ CFU/mL) was dropped onto the center of sterilized carboxymethyl cellulose membranes overlaid on PDA medium and incubated for 10 days in the dark. The germinating conidia attached on the mycelia of the strains were removed by washing using sterile water. In addition, the cellophane membranes were rinse twice using double distilled water (ddH_2_O). Then the mycelia of each strain penetrating the carboxymethyl cellulose membranes were harvested and collected for RNA isolation for transcriptomic analysis. Three independent repeats were performed. Total RNA extraction and quality assessment, cDNA library construction, data assembly, sequence alignment to reference genomes, and unigene annotation were performed by Biomarker Technologies Co., Ltd. (Beijing, China) as described before (Li et al., 2017). Screening of differentially expressed genes was done using the screening criterion of DEGseq2 with a fold change of >2.0 and FDR <0.01 (Love et al., 2014). R/top GO (2.18.0) method was used for GO functionally significant enrichment analysis with a *p*-value <0.05 as the threshold. The ClusterProfiler (v3.4.4) software was used to statistically analyze the enrichment of differentially expressed genes in KEGG pathways. DEGs with a corrected *p*-value <0.05 were considered to be significantly enriched. In addition, KEGG pathways were visualized using R ggplot2 package (v3.4.4). The Benjamini–Hochberg correction method was applied to obtain significant *p*-values as previously proposed (Benjamini and Hochberg, 1995).

### 4.9 Chitin and β-1,3-glucan content determination

For determination of chitin content and β-1,3-glucan content, 50 μL of conidial suspension (1 × 10^7^ CFU/mL) of each strain was dropped into 50 mL of liquid CM medium and incubated at 25°C with shaking (200 rpm/min) for 5 days in the dark. The mycelia of each strain were harvested, thoroughly rinsed using sterilized ddH_2_O and immediately frozen in liquid nitrogen. Additionally, 10 μL of the conidia suspension (1 × 10^7^ CFU/mL) of each strain was dropped onto the center of sterilized carboxymethyl cellulose membranes overlaid on PDA medium and incubated for 7 days in the dark. The germinating conidia attached on the mycelia of each strain were removed by washing using sterile water. In addition, the cellophane membranes were rinse twice using ddH_2_O. Then the mycelia of each strain on the carboxymethyl cellulose membranes were harvested, and then frozen in liquid nitrogen. The chitin content in the frozen fungal samples was determined as previously described (Lee et al., 2005). The β-1,3-glucan content in the frozen fungal samples was determined by phenol-sulphuric acid method according to previous report (Moya and Dewar, 1956). Three independent repeats were performed.

### 4.10 Statistical analysis

In all experiments, three independent repeats were performed. Data were statistically analyzed on R environment version (4.3.2). The ggplot2 package was employed to generate bar plots which represented mean ± standard deviation from three independent repeats. Significance differences between treatments were analyzed by one-way analysis of variance (ANOVA) using Duncan’s multiple range tests (DMRT) implemented on agricolae package. Significant differences between treatments were determined at *p*-value of 0.01 or 0.05.

## Data Availability

The datasets presented in this study can be found in online repositories. The names of the repository/repositories and accession number(s) can be found in the article/Supplementary material.

## Footnotes

^1^ https://fungi.ensemble.org/Verticilliumdahliae/Index

^2^ https://github.com/CJ-Chen/TBtools/releases

^3^ https://ncbi.nlm.nih.gov

^4^ https://www.smart.Emble-heidelbeg.de

^5^ http://dog.biocuckoo.org

^6^ https://cbs.dtu.dk/services/TMHMM/

^7^ https://itol.embl.de
