# Supplementary figures and images for: VdPAT1 encoding a pantothenate transporter protein is required for fungal growth, mycelial penetration and pathogenicity of Verticillium dahliae
Source: Front Microbiol. 2025 Jan 17;15:1508765. doi: 10.3389/fmicb.2024.1508765 (PMC11783681; doi:10.3389/fmicb.2024.1508765)

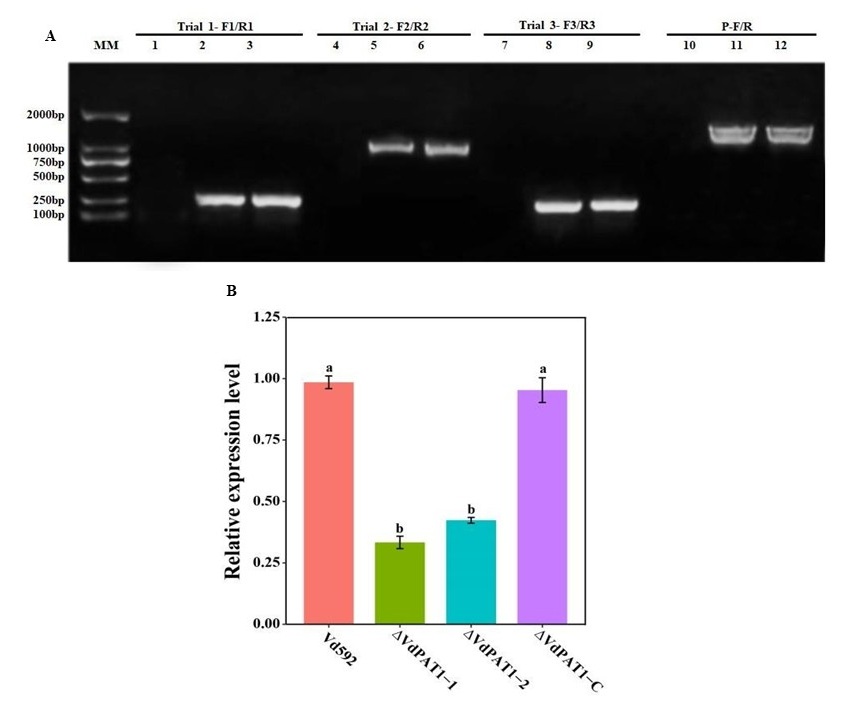

Supplement: Supplementary file 1 [file Image_1.jpeg]

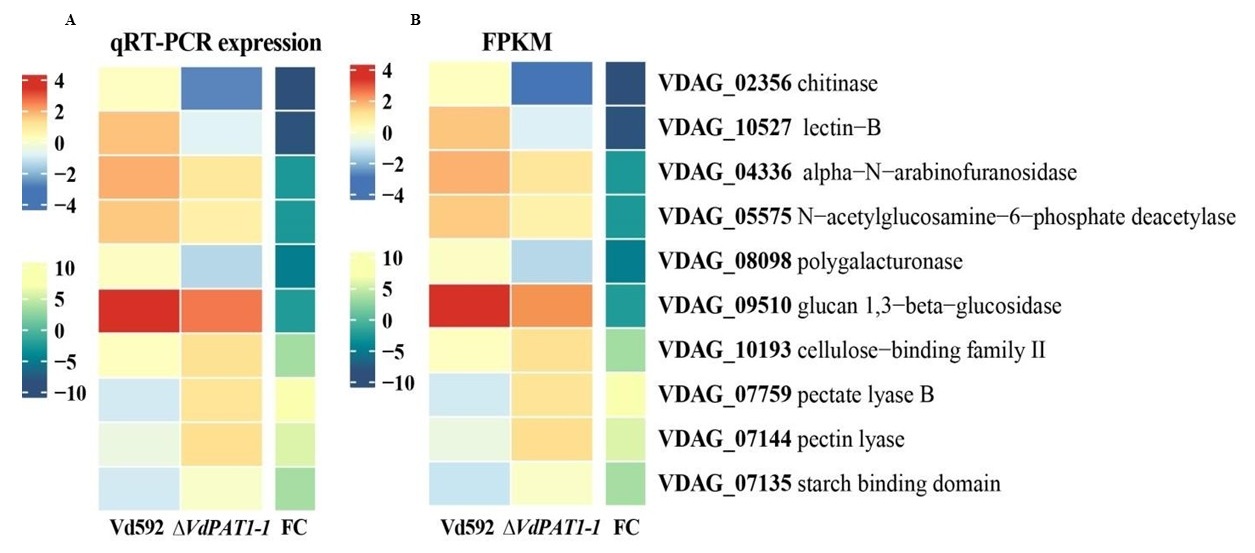

Supplement: Supplementary file 2 [file Image_2.jpeg]

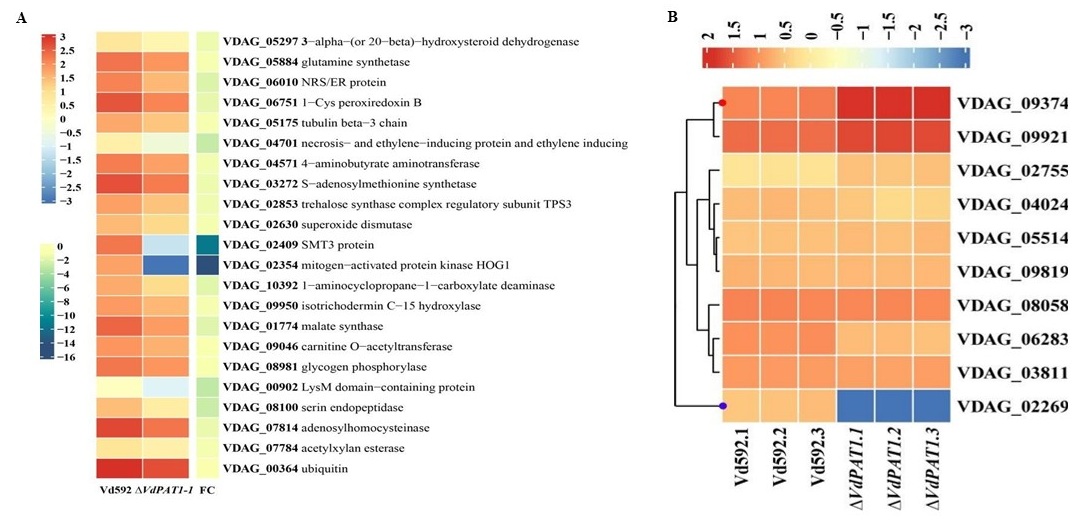

Supplement: Supplementary file 3 [file Image_3.jpeg]
