# Supplementary material for: VdPAT1 encoding a pantothenate transporter protein is required for fungal growth, mycelial penetration and pathogenicity of Verticillium dahliae
Source: Front Microbiol. 2025 Jan 17;15:1508765. doi: 10.3389/fmicb.2024.1508765 (PMC11783681; doi:10.3389/fmicb.2024.1508765)
Supplement: Supplementary file 4 [file Supplementary_file_1.docx]

Supplementary Material

# Supplementary Figures and Tables

## Supplementary Figures

**Supplementary Figure 1.** PCR and qRT-PCR confirmation for the *VdPAT1* deletion mutants and complementary strain.

(**A**) PCR confirmation of the Δ*VdPAT1* mutants (Δ*VdPAT1-1* and Δ*VdPAT1-2*) and complementary strain (Δ*VdPAT1-C*). Lanes 1-3 were PCR amplification results using Trial-F1/R1 as primers; lanes 4-6 were PCR amplification results using Trial-F2/R2 as primers; lanes 7-9 were PCR amplification results using Trial 3-F3/R3 as primers and lanes 10-12 were PCR amplification results using P-F/R as primers. MM represents (DS2000 molecular marker). Lanes 1, 4 and 7 were the PCR results using Vd592 DNA as template*.* Lanes 2, 3, 5, 6, 8, 9 and 10 were the PCR results using DNA from Δ*VdPAT1* mutants (Δ*VdPAT1-1* and Δ*VdPAT1-2*) as template. Lanes 11 and 12 were the PCR results using complementary strain (Δ*VdPAT1-C*) DNA as template.

(**B**) The expression level of *VdPAT1* in the *VdPAT1* deletion mutants and complementary strain detected by qRT-PCR.

**Supplementary Figure 2.** Validation of RNA-Seq results by qRT-PCR.

(**A**)The qRT-PCR results showing the expression level of 10 randomly selected DEGs in Vd592 and Δ*VdPAT1-1.*

(**B**) A heatmap based on RNA-seq data showing the expression level of 10 randomly selected DEGs in Vd592 and Δ*VdPAT1-1.*

**Supplementary Figure 3.** Heatmaps of HPI genes and other pantothenate transporter genes in *V. dahliae*

(**A**) A heatmap showing the expression level of 22 PHI genes

(**B**) A heatmap showing the expression level of 10 pantothenate transporter genes identified in *V. dahliae*.

## Supplementary Table

**Supplementary Table 1.** Table S1 All the primers used in this study.
